# The crosstalk between microbiota and metabolites in AP mice: an analysis based on metagenomics and untargeted metabolomics

**DOI:** 10.3389/fcimb.2023.1134321

**Published:** 2023-08-09

**Authors:** Qi Zhou, Xufeng Tao, Fangyue Guo, Yutong Zhu, Yu Wu, Hong Xiang, Dong Shang

**Affiliations:** ^1^ Laboratory of Integrative Medicine, First Affiliated Hospital of Dalian Medical University, Dalian, China; ^2^ Institute (College) of Integrative Medicine, Dalian Medical University, Dalian, China; ^3^ Department of Pharmacy, First Affiliated Hospital of Dalian Medical University, Dalian, China; ^4^ Department of General Surgery, Pancreatic-Biliary Center, First Affiliated Hospital of Dalian Medical University, Dalian, China

**Keywords:** acute pancreatitis, gut microbiota, metabolites, metabolomics, metagenomics

## Abstract

**Background and purpose:**

Microbiome dysfunction is known to aggravate acute pancreatitis (AP); however, the relationship between this dysfunction and metabolite alterations is not fully understood. This study explored the crosstalk between the microbiome and metabolites in AP mice.

**Methods:**

Experimental AP models were established by injecting C57/BL mice with seven doses of cerulein and one dose of lipopolysaccharide (LPS). Metagenomics and untargeted metabolomics were used to identify systemic disturbances in the microbiome and metabolites, respectively, during the progression of AP.

**Results:**

The gut microbiome of AP mice primarily included Firmicutes, Bacteroidetes, Actinobacteria, and Proteobacteria, and “core microbiota” characterized by an increase in Proteobacteria and a decrease in Actinobacteria. The Kyoto Encyclopedia of Genes and Genomes (KEGG) analysis found that significantly different microbes were involved in several signaling networks. Untargeted metabolomics identified 872 metabolites, of which lipids and lipid-like molecules were the most impacted. An integrated analysis of metagenomics and metabolomics indicated that acetate kinase (*ackA*) gene expression was associated with various gut microbiota, including *Alistipes*, *Butyricimonas*, and *Lactobacillus*, and was strongly correlated with the metabolite daphnoretin. The functional gene, *O*-acetyl-L-serine sulfhydrylase (*cysK*), was associated with *Alistipes*, *Jeotgalicoccus*, and *Lactobacillus*, and linked to bufalin and phlorobenzophenone metabolite production.

**Conclusion:**

This study identified the relationship between the gut microbiome and metabolite levels during AP, especially the *Lactobacillus-, Alistipes-*, and *Butyricimonas*-associated functional genes, *ackA* and *cysK*. Expression of these genes was significantly correlated to the production of the anti-inflammatory and antitumor metabolites daphnoretin and bufalin.

## 1 Introduction

Acute pancreatitis (AP) is one of the most common gastrointestinal diseases observed in clinical practice (Lee and Papachristou, 2019; Nassar and Qunibi, 2019). Reduced diversity in the gut microbiome is associated with AP pathogenesis in both humans and animals (Zheng et al., 2019). Li et al. found a significantly altered bacterial microbiome in the blood of patients with severe AP, including a higher abundance of Bacteroidetes and Firmicutes, and a lower abundance of Actinobacteria (Li et al., 2018). Obvious alterations in the predominant fecal microbes were also observed in AP patients with mild and severe disease (Zhu et al., 2019; Mei et al., 2022). As a result, the gut microbiome is now considered a critical factor involved in AP pathogenesis (Huang et al., 2017). Metagenomic sequencing is being increasingly used in research studies, allowing for a deeper characterization of microbiome complexity, identification of a higher number of species in each sample, and the definition of metabolic pathways and gene function at the molecular level (Ye et al., 2019). Metagenomics has been used to identify the etiology of AP from biological samples, survey taxonomic classification, and identify underlying disease mechanisms.

Gut microbiota modulate host health and disease using multiple metabolic pathways and immune–inflammatory axes (Nicholson et al., 2012). Some bacterial metabolites are not restricted to the intestine and circulate to affect the physiology and pathology of distant organs (Ticinesi et al., 2018). For example, acetate generated by *Parabacteroides* can prevent heparinase-induced AP progression by decreasing neutrophil infiltration (Lei et al., 2021). Although several studies have analyzed short-chain fatty acid- and bile acid-producing bacteria, little is known about how non-classical metabolites, produced by gut microbes, modulate AP. As the critical roles of the gut microbiome and their metabolites have emerged, it has become more important to identify new microbe–metabolite combinations and determine how they are linked to the pathogenesis of AP.

This study used metagenomics and untargeted metabolomics to investigate the relationship between disturbances in the gut microbiome and the development of fecal metabolic disorders in the AP model. A deeper understanding of the crosstalk between gut microbes and metabolites could be a prerequisite for characterizing the underlying mechanisms by which metabolites affect the course of this disease.

## 2 Materials and methods

### 2.1 Animals and ethics approval

Male C57BL/6J mice (10 weeks of age) were provided by the Laboratory Animal Center at Dalian Medical University. Mice were raised under specific pathogen-free conditions with a controlled temperature (25°C ± 1°C) and free access to drinking water and standard laboratory feed. The experimental model of AP was induced by hourly intraperitoneal injections of cerulein (100 μg/kg) and one injection of lipopolysaccharide (LPS, 10 mg/kg). Mice in the control (Ctrl) group received similar injections of physiological saline. The animal experiments complied with Animal Research: Reporting of in vivo Experiments (ARRIVE) guidelines, were conducted in accordance with the National Research Council’s Guide for the Care and Use of Laboratory Animals, and were approved by the Institutional Animal Ethics Committee at Dalian Medical University (No. AEE21019).

### 2.2 Serum enzyme assays

Serum was obtained after centrifuging blood samples at 2,500 revolutions/minute for 20 minutes at 4°C. Amylase and lipase levels in the serum were evaluated using commercial reagents purchased from Nanjing Jiancheng Bioengineering Institute (Nanjing, China). The detailed operating procedures followed the manufacturer’s instructions.

### 2.3 Quantitative polymerase chain reaction assays

After sacrificing the mice, the pancreases were harvested and immediately frozen in liquid nitrogen. Pancreas samples were ground with 1 mL of TRIzol^®^ (Accurate Biotechnology, China) and total RNA was extracted after the successive addition of chloroform and isopropanol. cDNA synthesis was conducted in accordance with the manufacturer’s instructions. The SYBR™ Green kit (Accurate Biotechnology, China) was used to examine gene expression using a BioTek Cytation 3 instrument (Hangzhou, China). All primers were purchased from Sangong Biotech Co. (Shanghai, China) and are shown in **Table 1**.

**Table 1:** Primer sequences used for quantitative polymerase chain reaction.

Gene	NM_number	Primers (5′–3′)
β-actin	NM_007393.5	Forward: CTACCTCATGAAGATCCTGACC Reverse: CACAGCTTCTCTTTGATGTCAC
IL-1β	NM_008361.4	Forward: CACTACAGGCTCCGAGATGAACAAC Reverse: TGTCGTTGCTTGGTTCTCCTTGTAC
IL-6	NM_031168.2	Forward: CTCCCAACAGACCTGTCTATAC Reverse: CCATTGCACAACTCTTTTCTCA
TNF-α	NM_013693.3	Forward: ATGTCTCAGCCTCTTCTCATTC Reverse: GCTTGTCACTCGAATTTTGAGA

### 2.4 Hematoxylin and eosin staining

Fresh pancreas tissue was fixed in a formaldehyde solution and embedded in paraffin wax. After deparaffinization and rehydration, sections with a thickness of approximately 5 μm were sliced for staining with hematoxylin and eosin (HE). Images were captured using the digital slide scanner Pannoramic MIDI, from 3DHISTECH Ltd (Budapest, Hungary).

### 2.5 Metagenomics

Fresh fecal samples were collected from mice and immediately frozen in liquid nitrogen. The Illumina PE150 was used for sequencing. To ensure data accuracy and reliability, the original data were cleaned and quality assessed, and an information analysis was performed.

### 2.6 Untargeted metabolomics

Fecal samples were thawed slowly at 4°C, and then metabolites were separated using a pre-cooled methanol/acetonitrile/aqueous solution [2: 2: 1, v/v (volume to volume)]. A mass spectrometric analysis was performed using Q Exactive™ series mass spectrometry (Thermo Fisher Scientific, Waltham MA, USA) in both positive and negative modes. Raw data, characterized using the ProteoWizard format, were converted into the mzXML format. XCMS software was used to perform peak alignment, time correction, and extraction of the peak area, and the metabolite structures were confirmed. Analysis of the experimental data was then conducted.

### 2.7 Integrated metagenomics and metabolomics analysis

To simplify the data, a principal coordinate analysis (PCoA) analysis was used to reflect differences in multidimensional data on a two-dimensional coordinate graph. Considering the non-normal distribution of the original data, a Spearman analysis was used to evaluate the correlation coefficient between significantly different gut microbes and metabolites. Using the results of metagenomic analysis, the primary contributing species for functional genes were obtained. Based on the relationship between species and functional genes and functional genes and metabolites, the multilevel regulatory relationships between species–functional genes–metabolites were explored.

### 2.8 Statistical analysis

For the statistical analysis of the metagenomics data, a principal component analysis (PCA) (R ade4 package) was used to compare the taxonomic and functional compositions among the samples. Wilcoxon (two groups) and linear discriminant analysis effect size (LEfSe) analyses were used to explore different species or functions at each level in the Ctrl and AP groups. For the untargeted metabolomics analysis, a multivariate data analysis was conducted using the R package (ropls; The R Foundation for Statistical Computing, Vienna, Austria). Variable importance in projection (VIP) scores > 1 and *q*-values < 0.05 were used as inclusion criteria to select significantly altered metabolites. All data were presented as the means ± SEM. Prism7 software (GraphPad Software Inc., San Diego, CA, USA) was used for the statistical analysis. A two-tailed unpaired Student’s *t*-test was used to analyze the differences. The *p*-values that were < 0.05 were considered statistically significant.

## 3 Results

### 3.1 Study design

**Figure 1:**
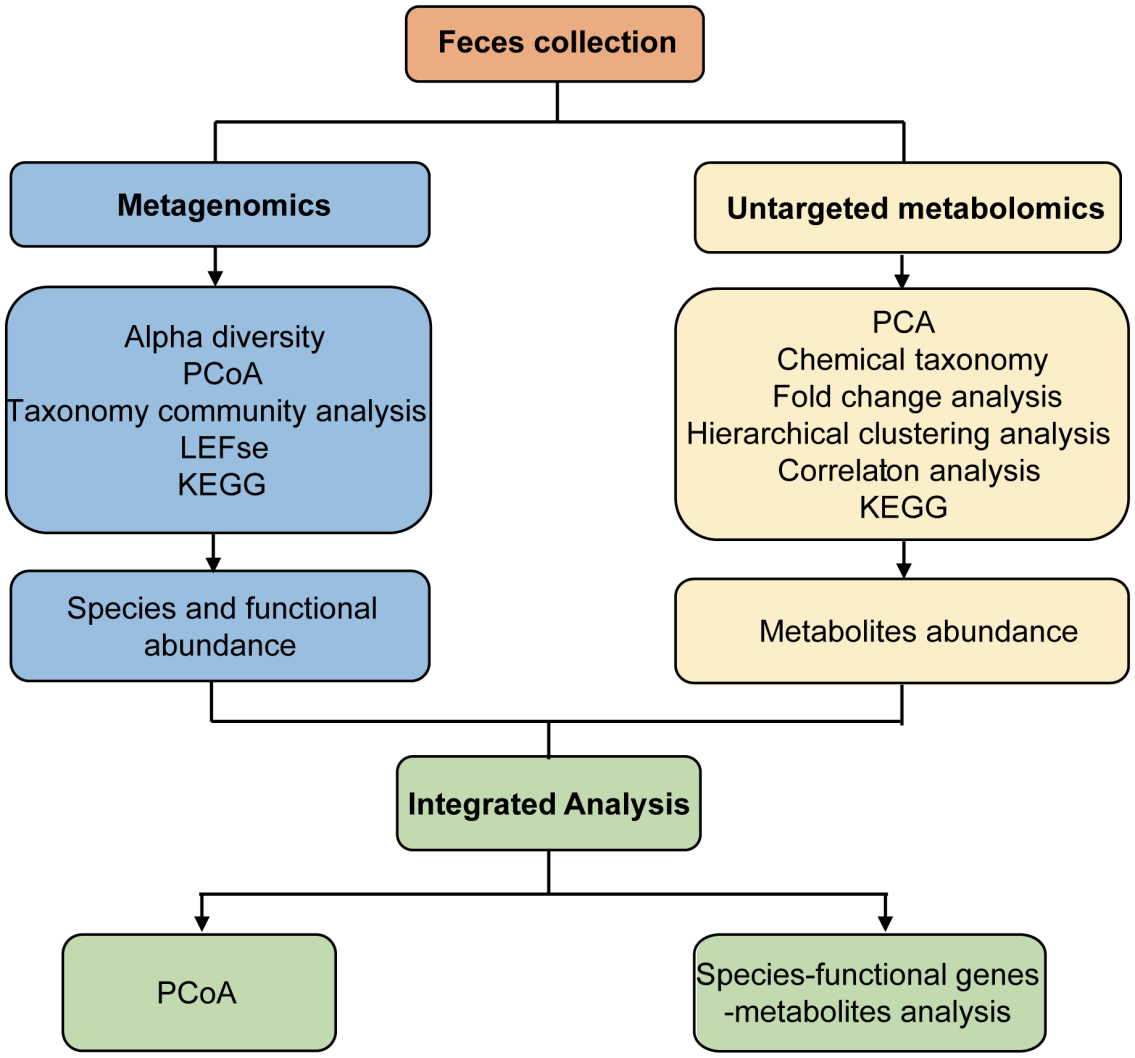
Study design and workflow.

Fresh fecal samples from Ctrl and AP mice were aseptically obtained (**Figure 1**). Metagenomics and untargeted metabolomics were used to detect different gut microbes and metabolites. In addition, differentially expressed gut microbiota-associated genes were identified using cluster, PCA, LEfSe, and metabolic pathway analyses. After the different metabolites were screened, clustered, and correlated, a Kyoto Encyclopedia of Genes and Genomes (KEGG) analysis was used for functional annotation. Finally, an integrated analysis of the metagenomics and metabolomics data was used to explain the association between different gut microbes and metabolites, helping to establish a logical relationship among microorganisms–metabolites–phenotypes during AP.

### 3.2 The successful establishment of an acute pancreatitis mouse model using cerulein combined with lipopolysaccharide

**Figure 2:**
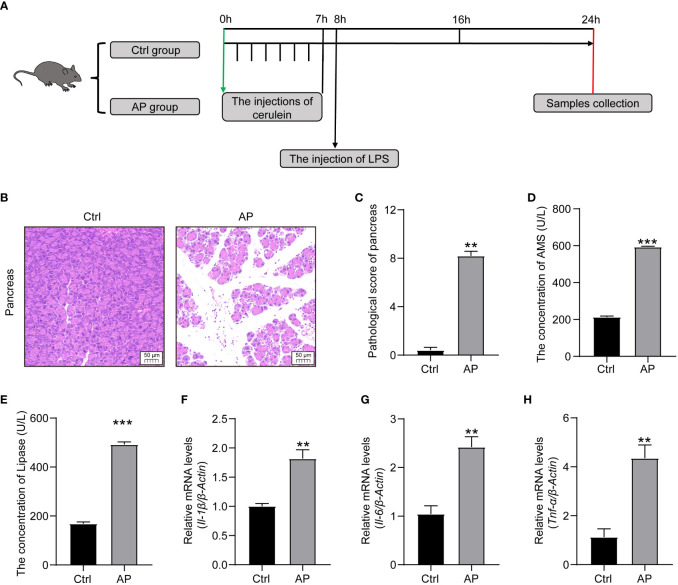
Establishment of the AP mouse model. **(A)** Schematic diagram of the experimental model. **(B)** Histopathological changes of the pancreas (HE, ×200). **(C)** Pancreatic histologic scores in the Ctrl and AP groups. **(D, E)** Amylase and lipase serum levels. **(F–H)** TNF-α, IL-1β, and IL-6 expression in the pancreas. Data are presented as the mean ± SEM; **P<0.01 vs. the Ctrl group, ***P<0.001 vs. the Ctrl group.

A schematic diagram of the experimental establishment of an AP mouse model is shown in **Figure 2A**. To evaluate whether or not the model was successfully constructed, pancreas histopathology and cytokine and serum enzyme levels (lipase and amylase) were assessed. Typical HE staining of the pancreas revealed obviously enlarged interlobular interspaces, inflammatory cell infiltration, and even acinar damage in the AP group. In addition, the pancreatic pathological scores were obviously higher and there was a marked increase in serum lipase and amylase levels in the AP group mice than those in the Ctrl group (**Figures 2B–E**). The production of proinflammatory cytokines, such as tumor necrosis factor alpha (TNF-α), interleukin 1 beta (IL-1β), and interleukin 6 (IL-6), in the pancreas was significantly higher in the AP mice than in the Ctrl mice (**Figures 2F–H**). These results indicate that AP mice had potent pancreatic and systematic inflammation, illustrating that the AP mouse model with cerulein combined with LPS had been successfully established.

### 3.3 The characteristic phenotype of the gut microbiome in acute pancreatitis mice

**Figure 3:**
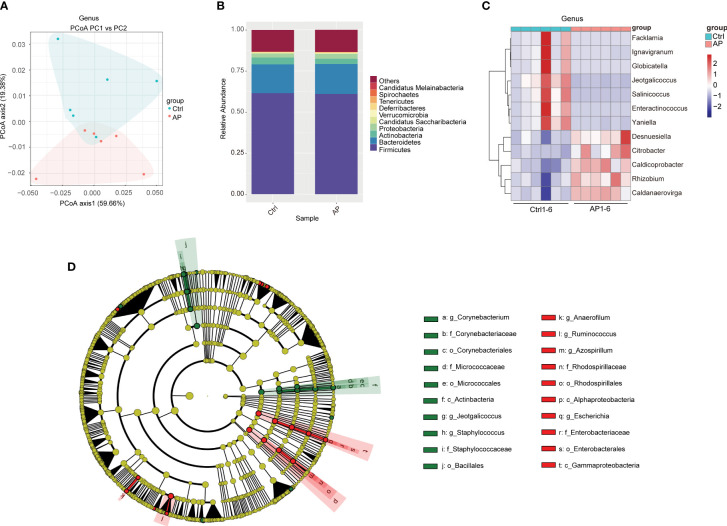
Differences in the gut microbiota of the Ctrl and AP groups. **(A)** PCoA analysis of the gut microbiota structure and abundance in the Ctrl and AP mice. **(B, C)** Relative abundance of the gut microbiota at the phylum and genus levels. **(D)** LEfSe analysis of the Ctrl and AP mice using a cladogram.

Alpha diversity analyses were used to measure microbiome richness and uniformity. According to the Chao1, abundance-based coverage estimator (ACE), Shannon, and Simpson indexes, there were no significant differences in the overall richness and uniformity of the gut microbiota in the AP and Ctrl groups (*P* > 0.05) (**Supplementary Figures 1A–D**). To conduct a beta diversity analysis, a PCoA was used to reflect the structure of the gut microbiota by assessing the sample distance between the two groups. The composition of the microbiome in the control and AP mice was well differentiated at the species level (**Figure 3A**).

To conduct a taxonomic community analysis of the AP mice, the structure and relative abundance of the gut microbiota were investigated at both the phylum and the genus levels. Firmicutes, Bacteroidetes, Actinobacteria, and Proteobacteria were the dominant bacteria at the phylum level in both the AP and the Ctrl groups (**Figure 3B**). The AP mice had a noticeably lower abundance of Actinobacteria and Bacteroidetes, and a higher abundance of Proteobacteria and Firmicutes, suggesting that Proteobacteria and Firmicutes may be the “core microbiota” associated with AP. At the genus level, the relative abundances of *Facklamia*, *Ignavigranum*, *Globicatella*, *Jeotgalicoccus*, *Salinicoccus*, *Enteractinococcus*, and *Yaniella* were all lower in the AP mice than in the Ctrl mice, whereas the levels of *Desnuesiella*, *Citrobacter*, *Caldicoprobacter*, *Rhizobium*, and *Caldanaerovirga* were higher (**Figure 3C**). The corresponding cladograms in the AP and Ctrl mice are shown in **Figure 3D**. The relative quantities of *Corynebacterium, Staphylococcus, Jeotgalicoccus*, Firmicutes bacterium CAG 822, and *Clostridium* sp. CAG 628 were higher in the Ctrl group, whereas the relative quantities of *Alistipes* sp. Marseille P2431, *Alistipes* sp. CAG 53, *Alistipes shahii*, *Clostridium clariflavum*, *Anaerofilum*, and *Azospirillum* were significantly higher in the AP mice.

### 3.4 Enrichment analysis of functional genes associated with the gut microbiome of acute pancreatitis mice

**Figure 4:**
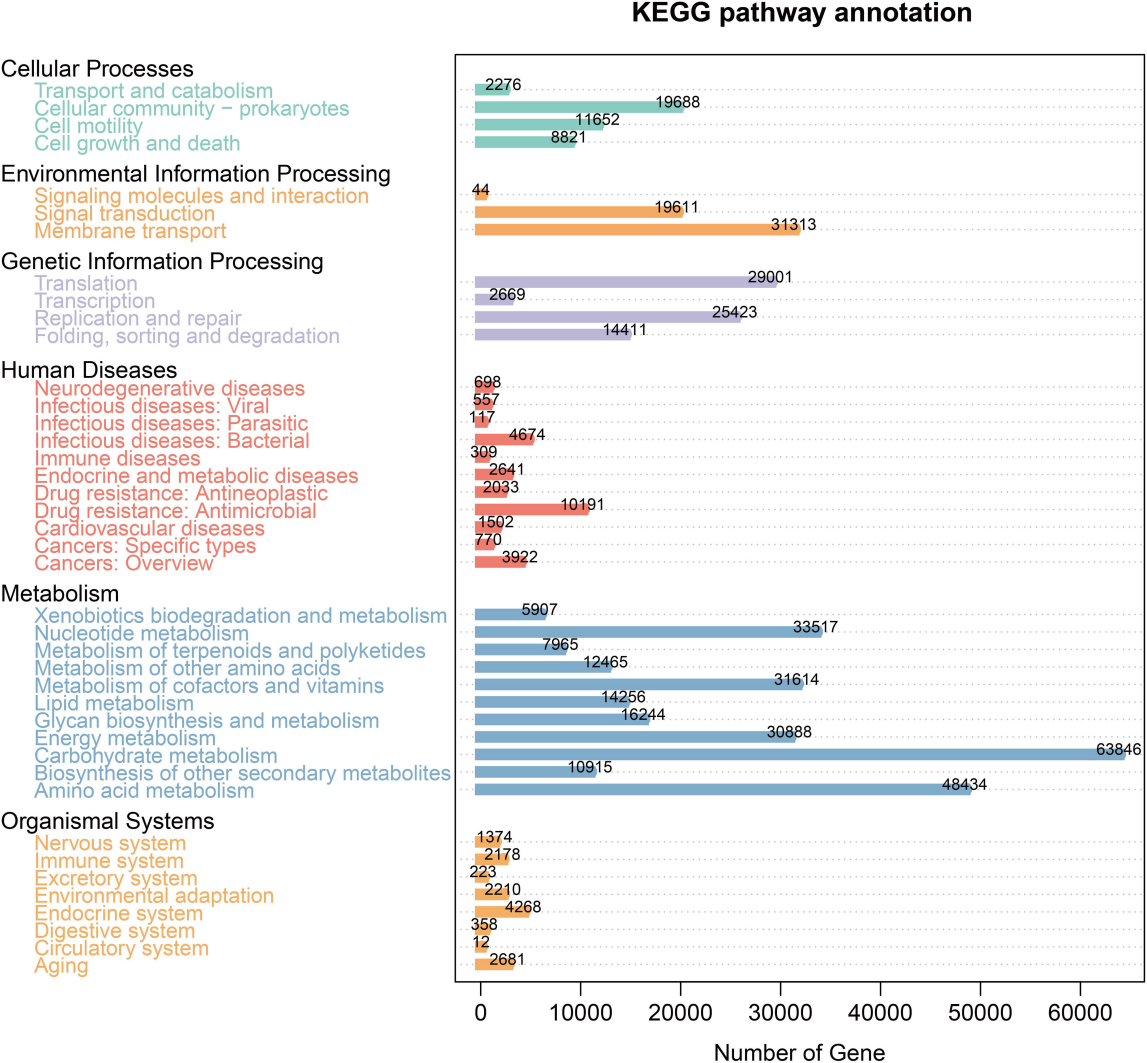
KEGG pathway enrichment.

The KEGG database analysis was used to explore the important molecular networks and biological pathways. Different microbiota were found to be associated with several signaling pathways primarily affiliated with metabolism (i.e., amino acid, carbohydrate, and lipid) and environmental information processing (i.e., signal transduction and membrane transport) (**Figure 4**). These results provided significant information about the functional genes associated with the gut microbiota of AP mice.

### 3.5 The characteristic phenotype of metabolites in acute pancreatitis mice

**Figure 5:**
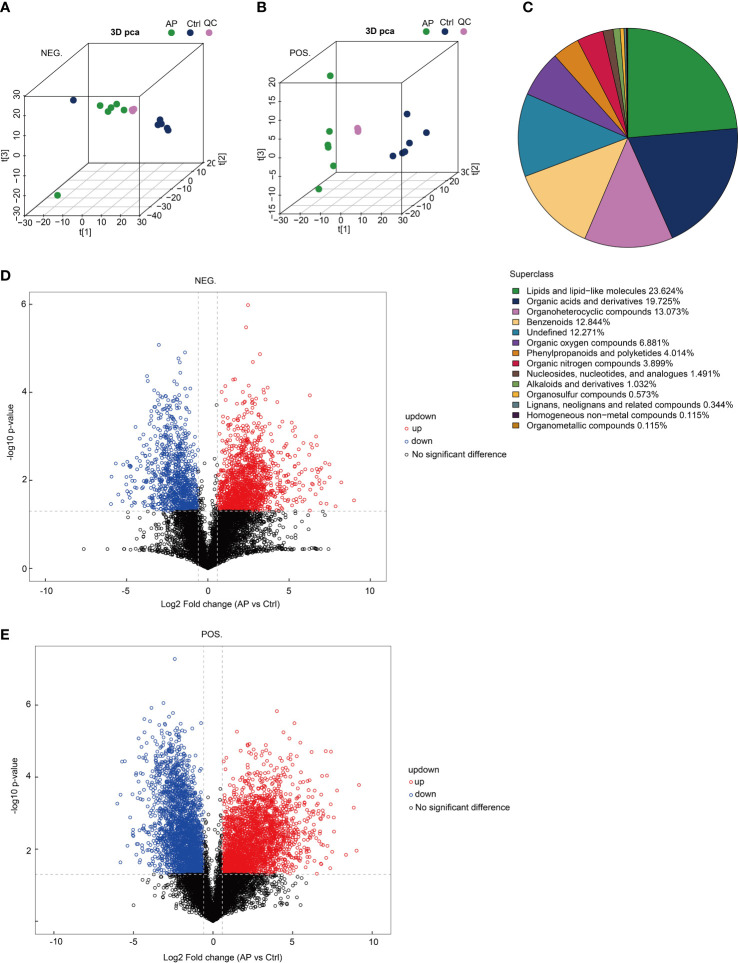
Analysis of untargeted metabolomics. **(A, B)** The reproducibility of the experiment was evaluated using a population sample principal component analysis (PCA-QC). **(C)** The proportion of identified metabolites in each chemical class. **(D, E)** Volcano plots show fold changes in metabolite levels.

To determine the reproducibility of the experiment, quality control (QC) samples were analyzed using PCA-QC. The t[1] and t[2] were found to represent principal components 1 and 2, respectively (**Figures 5A, B**). These results indicated that the degree of aggregation of QC samples in the positive and negative modes nearly overlapped, suggesting that the reproducibility of the experiment was strong. A total of 872 metabolites were screened and confirmed by applying untargeted methods and all (combined with the positive and negative models) were specifically grouped according to their chemical classification. The attribution information was classified and statistically calculated, and the proportion of various metabolites was determined (**Figure 5C**). The undefined part included metabolites that could not be chemically classified. The metabolites primarily belonged to several chemical classes, including lipid and lipid-like molecules and organic acids and their derivatives. Volcano plots were created to illustrate the differences and average intensity change ratios of differential metabolites [fold change (FC) > 1.5 or FC < 0.67; *q* < 0.05] (**Figures 5D, E**). A total of 872 metabolites, which were significantly differentially produced, were confirmed using untargeted metabolomics, of which 486 metabolites were upregulated and 386 metabolites were downregulated.

### 3.6 Bioinformatics analysis of differential metabolites

**Figure 6:**
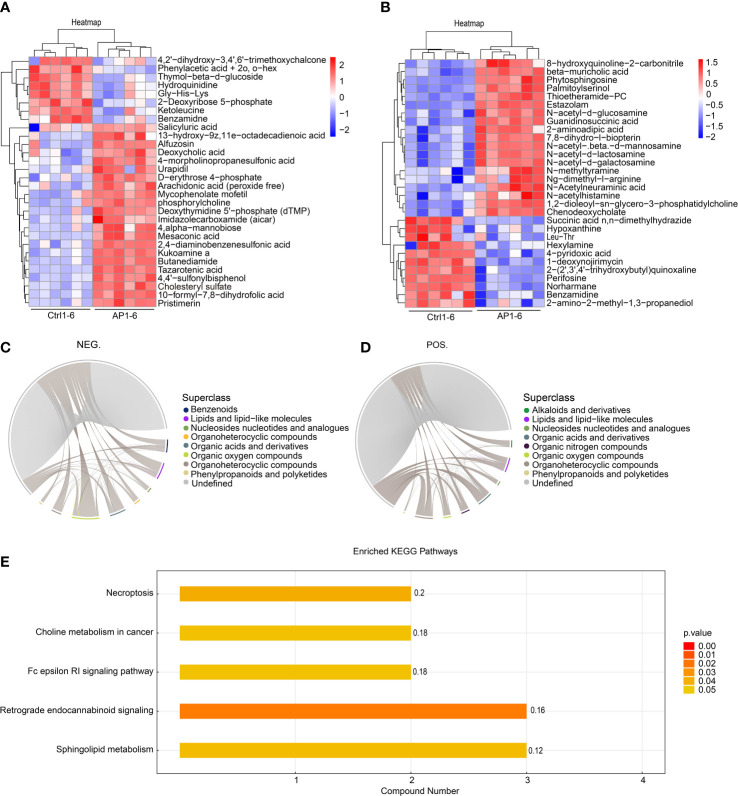
Bioinformatic analysis of differential metabolites. **(A, B)** A heatmap shows the top 30 differentially produced metabolites. **(C, D)** Chord diagrams show the coregulatory relationships between the various metabolites. **(E)** KEGG analysis of enrichment pathways.

The top 30 differentially produced metabolites were visualized using a heat map (**Figures 6A, B**). Although the levels of hydroquinidine, benzamidine, and perifosine were lower in the AP group, the levels of cholesteryl sulfate, pristimerin, and beta-muricholic acid were higher. After screening significantly differentially produced metabolites (i.e., with a VIP score > 1 and a *p*-value < 0.05), a subsequent bioinformatic analysis was conducted using a correlation analysis. To visualize the coregulatory relationships between the various metabolites, a chord diagram was developed (**Figures 6C, D**).

The different metabolites screened using the positive and negative models were combined prior to annotation and analysis. Enrichment analysis of the KEGG pathway is shown in **Figure 6E**. The findings illustrated that significantly differentially produced metabolites were mainly involved in the signaling pathways associated with necroptosis, choline metabolism during cancer, Fc epsilon RI signaling, retrograde endocannabinoid signaling, and sphingolipid metabolism.

### 3.7 Integrated metagenomics and metabolomics analysis

**Figure 7:**
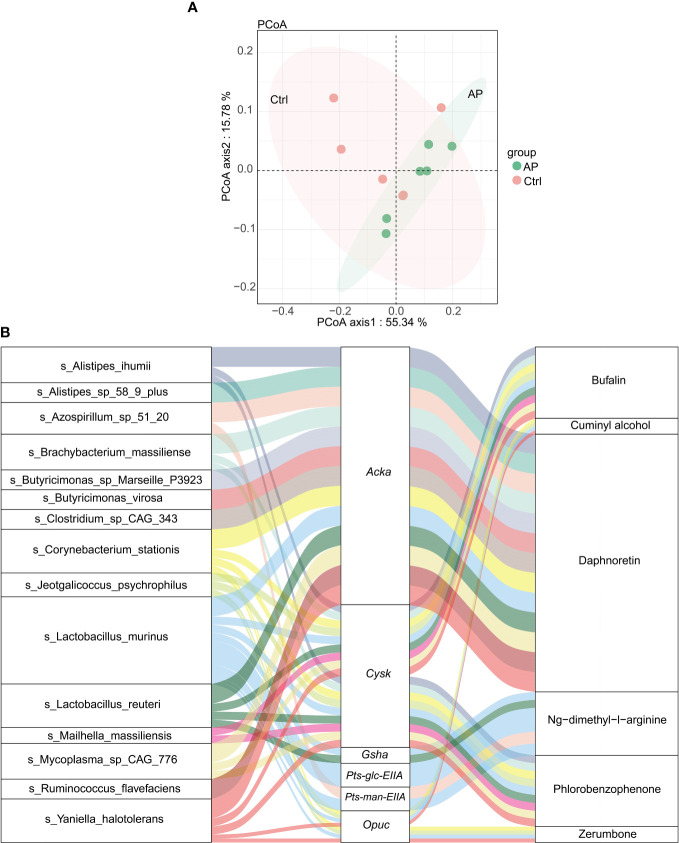
Integrated analysis of metagenomics and metabolomics. **(A)** The PCoA map. **(B)** A Sankey diagram showing the correlation of species–functional genes–metabolites.

Using the abovementioned results, integrated metagenomics and metabolomics analyses were performed to get a better understanding of the relationship between gut microbiota and metabolites. The more similar the community composition of the samples, the closer they were on the PCoA map (**Figure 7A**).

After calculating the significance using a Spearman analysis, a heat map was created to illustrate the correlation coefficient between the differential microbiota and metabolites. Significantly different genera are displayed in rows, and significantly different metabolites are displayed in columns (**Supplementary Figure 2**). For example, levels of *trans*-ferulic acid were clearly higher in the AP group than in the Ctrl group. The levels of *trans*-ferulic acid correlated positively with the bacteria *Corynebacterium stationis, Yaniella halotolerans, Lactobacillus apodemi, Alistipes ihumii, Butyricimonas* sp. Marseille_P3923*, Butyricimonas virosa*, Bacteroidales bacterium 55 9, and *Alistipes* sp. 58 9 plus. However, there was no significant association between the gut microbiota and hypoxanthine/succinic acid/cholesteryl sulfate.

To visualize the correlation between species, functional genes, and metabolites, a Sankey diagram was constructed using a Pearson correlation analysis (**Figure 7B**). The left column represents the species, the middle column illustrates the functional genes, and the right column shows the metabolites. The left connection lines represent contributing species for functional genes. The lines from the functional genes to the metabolites indicate that they are significantly correlated (|R| > 0.8, *p* < 0.05). The expression of the acetate kinase (ackA)gene was associated with various gut microbiota, including *A. ihumii*, *Alistipes* sp. 58 9 plus, *Azospirillum* sp. 51 20, *Brachybacterium massiliense*, *Butyricimonas* sp. Marseille P3923, *Butyricimonas virosa*, *Clostridium* sp. CAG_343, *C. stationis*, *Lactobacillus murinus*, *Lactobacillus reuteri*, *Mycoplasma* sp. CAG 776, *Ruminococcus flavefaciens*, and *Yaniella halotolerans*. The expression of *Acka* was correlated strongly with the metabolite daphnoretin. In addition, the expression of the functional *cysK* gene was correlated with *A. ihumii*, *B. massiliense*, *C. stationis*, *Jeotgalicoccus psychrophilus*, *L. smurinus*, *L. reuteri*, *Mailhella massiliensis*, *Mycoplasma* sp. CAG 776, and *Y. halotolerans*, and was associated with bufalin metabolites.

## 4 Discussion

There is a growing corpus of evidence that indicates that the composition of the gut microbiome differs between healthy and diseased states, and an imbalanced microbiome may contribute to the pathophysiology of gastrointestinal diseases and extraintestinal disorders of the pancreas (Van den Berg et al., 2021). Pancreatic microbiota have also been detected in both normal and diseased states. Bacterial translocation to the pancreas can occur orally, via the portal circulation from the lower gastrointestinal tract and through the mesenteric lymph nodes (Thomas and Jobin, 2020). In addition to the relationship between the oral microbiome and pancreatic function, alterations in pancreatic functions can directly affect the profile of the gut microbiome by inducing changes in metabolite production. Impaired exocrine pancreatic function is the most prominent host factor affecting the composition and diversity of the human intestinal microbiome (Frost et al., 2019; Girdhar et al., 2022).

Gut microbiota-derived metabolites have an important impact on AP disease progression. Some classes of metabolites, such as bile acids and short-chain fatty acids, are shown to regulate inflammation and cause immune dysregulation associated with several diseases (Lavelle and Sokol, 2020; Su et al., 2022). By integrating metagenomics and metabolomics, the current study found that *cysK* was related to the metabolite bufalin. The *cysK* gene encodes *O*-acetylserine sulfhydrylase, a key enzyme involved in cysteine desulfurization by *Lactobacillus casei* (Bogicevic et al., 2012). The *cysK* pairs with *CymR*, a transcriptional regulator of cysteine metabolism by *Bacillus subtilis* (Tanous et al., 2008). Bufalin belongs to a family of lipid and lipid-like molecules, including steroids and steroid derivatives, which have several antitumor, anti-inflammatory, and analgesic effects (Fang et al., 2021; Su et al., 2021; Soumoy et al., 2022). Bufalin is shown to act as an antitumor immune modulator that promotes the polarization of tumor-infiltrating macrophages from an M2 to an M1 phenotype, activating effector T-cell immune responses to suppress hepatocellular carcinoma (Yu et al., 2022). In rheumatoid arthritis fibroblast-like synoviocytes (RAFLSs), bufalin treatment has a dose-dependent inhibitory effect on IL-1β-induced proliferation by downregulating mitogen-activated protein kinases (MAPKs) and nuclear factor kappa B (NF-κB), and suppressing apoptosis through a mitochondrial-dependent pathway (Chang et al., 2014). Bufalin also suppresses the inflammatory response by inhibiting NF-κB activity in asthmatic mice (Zhakeer et al., 2017).

The current study found that the expression of *ackA*, which correlates with the metabolite daphnoretin, was also associated with various gut microbiota. Several bacteria were shown to generate acetic acid in response to glucose and other easily metabolizable carbohydrates (Won et al., 2021). Phosphotransacetylase (*Pta*)-*ackA* was also the central acetate-producing pathway. *Pta* induced acetylphosphate from acetyl coenzyme A, whereas *ackA* induced acetate from acetylphosphate, promoting bacterial ATP production (Schütze et al., 2020). These data reveal a major role for the *Pta-ackA* pathway in bacterial fitness. Interestingly, the inactivation of *Pta-ackA* signaling significantly increased the rate of glucose consumption, inhibited bacterial growth, and resulted in the death of *Staphylococcus aureus*, suggesting that the *Pta-ackA* pathway is critical to bacterial survival (Sadykov et al., 2013). Several studies have shown that daphnoretin (coumarins and derivatives) has significant anti-inflammatory and antitumor effects (Gu and He, 2012; Yu et al., 2019; Xie et al., 2022). Daphnoretin can suppress IL-1β-induced chondrocyte apoptosis by inhibiting endoplasmic reticulum stress and NOD-, LRR- and pyrin domain-containing protein 3 (NLRP3) inflammasome activation and suppressing osteoarthritis (Zhou and Wang, 2022). Further study is required to explore the functions of *cysK* and *ackA* in bufalin and daphnoretin metabolism during AP.

This study sought to examine different gut microbial microbiota and their associated metabolites in order to identify the functions of gut microbial-derived metabolites during AP. Metagenomics illustrated a dramatic alteration of the gut microbiota, including an overgrowth of *Escherichia* in the AP group, and high levels of *Jeotgalicoccus* and Firmicutes bacterium in the Ctrl group. The significantly different microbiota were found to be involved in amino acid, carbohydrate, and lipid metabolism as well as environmental information processing, including signal transduction and membrane transport. Untargeted metabolomics revealed that differentially produced metabolites are involved in AP. The metabolic pathways were enriched in necroptosis and sphingolipid metabolism. Meanwhile, integrated metagenomics and metabolomics found that the expression of *ackA* was associated with various gut microbiota. In particular, *ackA* was tightly correlated with the metabolite, daphnoretin, whereas the expression of *cysK* was correlated with bufalin metabolites. These findings suggest that targeting gut microbial microbiota and their associated metabolites could have a promising clinical application in novel treatments for AP.

## Data availability statement

The data of Untargeted metabolomics presented in the study are deposited in the Metabolights repository, accession number MTBLS6831. The data of Metagenomics presented in the study are deposited in the European Nucleotide Archive (ENA) repository, accession number PRJEB589.

## Ethics statement

The animal study was reviewed and approved by the Institutional Animal Ethics Committee of Dalian medical University. Written informed consent was obtained from the owners for the participation of their animals in this study.

## Author contributions

HX, DS, and XT developed and designed the study and revised the manuscript. QZ conducted the experimental work, acquired and analyzed the data, and wrote the first draft of the manuscript. FG, YW, and YZ contributed to the experimental work. All authors contributed to the article and approved the submitted version.
