# Supplementary material for: The crosstalk between microbiota and metabolites in AP mice: an analysis based on metagenomics and untargeted metabolomics
Source: Front Cell Infect Microbiol. 2023 Aug 9;13:1134321. doi: 10.3389/fcimb.2023.1134321 (PMC10446838; doi:10.3389/fcimb.2023.1134321)
Supplement: Supplementary file 1 [file DataSheet_1.docx]

Supplementary Material

**The Crosstalk between Microbiota and Metabolites in AP Mice Based on Metagenomics and Untargeted Metabolomics**

Qi Zhou^1,2#^, Xufeng Tao^3#^, Fangyue Guo^1,2^, Yutong Zhu^1,2^, Yu Wu^3^, Hong Xiang^1*^, Dong Shang^1, 2, 4*^

^1^ Laboratory of Integrative Medicine, First Affiliated Hospital of Dalian Medical University, Dalian, China

^2^ Institute (College) of Integrative Medicine, Dalian Medical University, Dalian, China

^3^ Department of pharmacy, First Affiliated Hospital of Dalian Medical University, Dalian, China

^4^ Department of General Surgery, Pancreatic-Biliary Center, First Affiliated Hospital of Dalian Medical University, Dalian, China

^#^ The authors contributed same work to this paper and they are the co-first authors.

^*^Corresponding Author: Hong Xiang, E-mail: [xianghong@dmu.edu.cn](mailto:xianghong@dmu.edu.cn); Dong Shang, E-mail: [shangdong@dmu.edu.cn](mailto:shangdong@dmu.edu.cn)


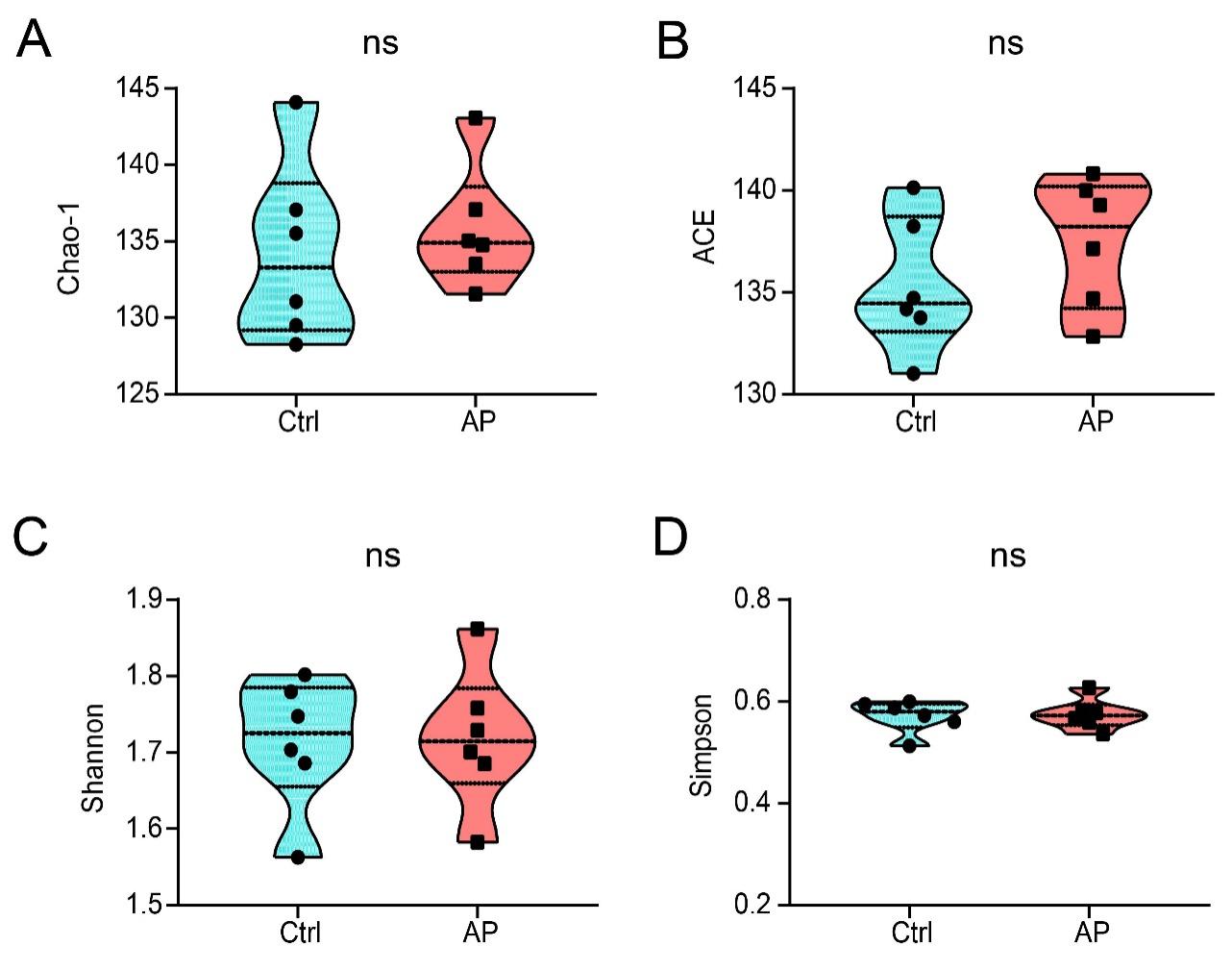


**Supplementary Figure 1. Alpha diversity between the Ctrl and AP groups.** (A-D) Alpha diversity using the Chao1, Observed species, Shannon, and Simpson indexes.


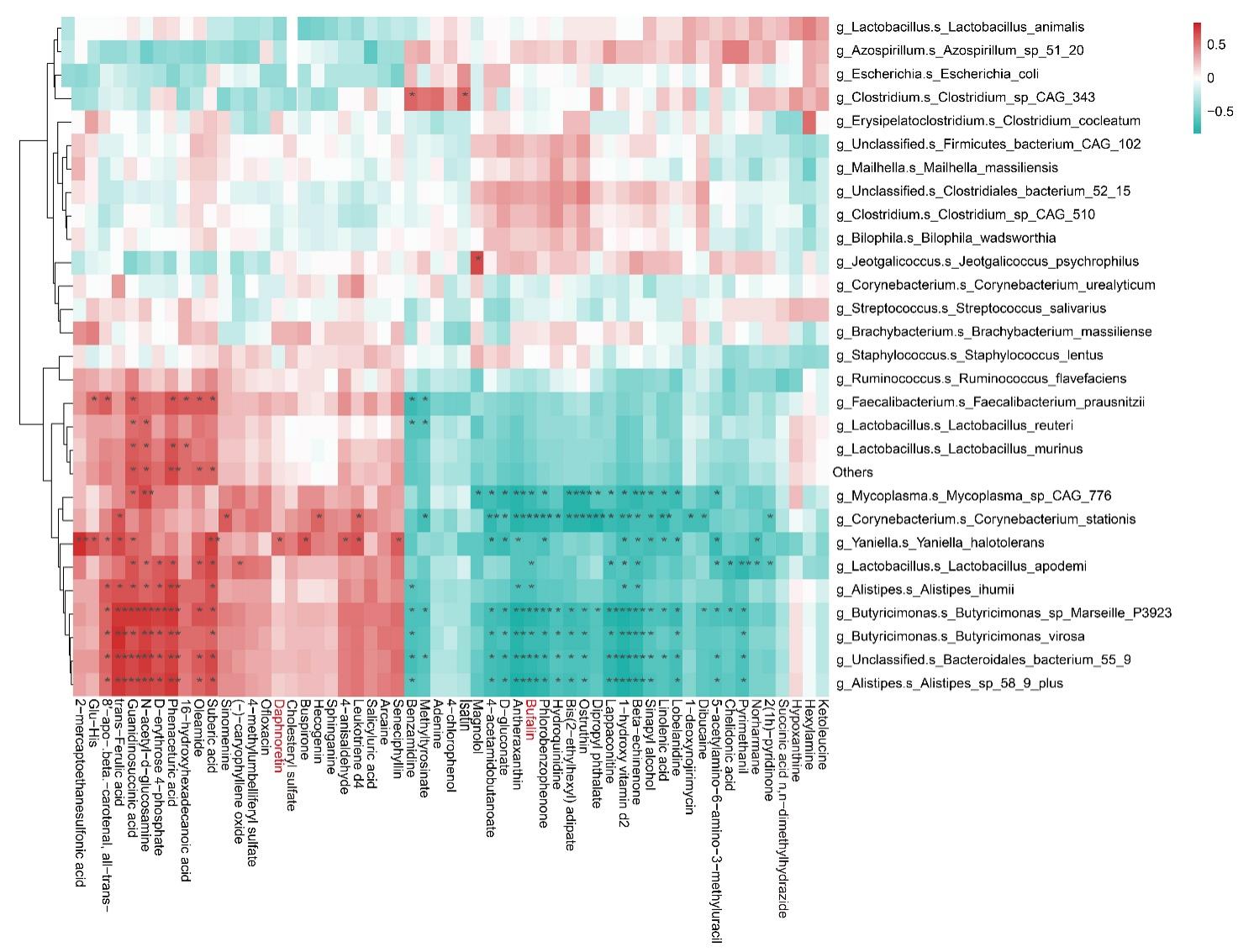
**Supplementary Figure 2. Correlation analysis.** A heat map shows the correlation coefficient between the differential microbiota and metabolites.
